# Validation of the Revised Collett–Lester Fear of Death Scale in a French Population

**DOI:** 10.3389/fpsyg.2021.736171

**Published:** 2021-10-25

**Authors:** Maeva Cuniah, Geneviève Bréchon, Nathalie Bailly

**Affiliations:** Department of Psychology, Psychology of the Various Stages of Life and Adaptation (PAVEA, EA 2114), University of Tours, Tours, France

**Keywords:** CL-FODS, validation, fear of dying and death, spirituality (MeSH), personality

## Abstract

Death and dying are processes that every human being encounters in his or her lifetime and perhaps the greatest loss an individual can suffer. In this sense, fear of death is regarded as a risk and maintaining factor of psychopathology. As such, effective and efficient measurement of this construct becomes a priority. While the Revised Collett-Lester Fear Of Death Scale (CL-FODS) is a brief, commonly used assessment, such a tool is lacking in French clinical practice. The present study aimed to adapt the revised CL-FODS in a general French sample and to determine its psychometric properties, namely its factorial structure, concurrent and convergent validity, and internal consistency. A sample of 590 participants responded to the French revised CL-FODS, as well as three instruments assessing death anxiety (DAS), neuroticism and spirituality (FACIT-Sp), to examine the internal consistency, validity and factorial structure of the scale. Both exploratory and confirmatory factor analysis confirmed a four-factor model: (1) One’s Own Death,” (2) The Death of Others, (3) The Dying of Others, and (4) One’s Own Dying. Five items did not load on these four factors, suggesting that the revised CL-FODS might require further psychometric refinement. The revised CL-FODS showed good internal consistency. The scale was found to be significantly associated with the Death Anxiety Scale. When the appropriate psychometric characteristics are taken into account, this scale can be used in clinical and research settings to assess death concerns in French society.

## Introduction

In a context in which the relationship with death has seen a profound transformation in our Western society, the question of death has long been repressed, and removed from the repertoire of life. In recent years, notably with the development of palliative care which encourages the acceptance of death, giving it a place in life, and supports people in facing the fears that it raises, there has been a growing interest in this long taboo subject in disciplines related to health and social sciences (Lockhart et al., 2001; Neimeyer et al., 2004; Solberg and Gamlund, 2016; Ding et al., 2020). The reflection on fear of death or to related terms has gradually given place to empirical studies that have assessed attitudes toward death and their socio-psychological correlates, structure and measurement, and applications of anxiety of death (Neimeyer et al., 2004). In the international literature, a number of previous assessment scales have approached fear of death as a unidimensional construct (Templer, 1970; Chow and Henry, 2017). However, at present, research highlights that fear of death includes several dimensions (Neimeyer, 1994; Abdel-Khalek, 1998; Wittkowski, 2001; Nyatanga and de Vocht, 2006; Roshani, 2012). The Collett–Lester Fear of Death Scale (CL-FODS, Collett and Lester, 1969) is probably one of the most popular multi-dimensional instruments used for research and clinical practice (Templer, 1970; Lo et al., 2011; Carlozzi et al., 2016). This scale differentiates two crucial aspects involved in death: (1) the fear of death versus the fear of dying and (2) the fear of one’s own death versus the fear of the death of others. By itself, it encompasses four subscales: “One’s own death,” “One’s own dying,” “Death of others” and “Dying of others.” The original CL-FODS highlighted a four-factor structure with good internal consistency. Since its first version (Collett and Lester, 1969), the CL-FODS has been revised reducing the number of items from 36 to 28 with an equal number of items in each subscale (seven-item subscales – Lester, 1990). The revised CL-FODS demonstrated acceptable reliability with good temporal stability and high internal consistency as well as suitable concurrent validity (Mooney and O’Gorman, 2001; Abdel-Khalek and Lester, 2004; Lester, 2004; Zeyrek and Lester, 2008). Studies using the CL-FODS have also investigated its association with a broad range of variables such as gender, age, religion, spirituality and personality Concerning gender, studies have yielded mixed results (Lester, 2015; Thiemann et al., 2015; Mohammadpour et al., 2018) while results indicate that older adults score lower than younger adults (Neimeyer et al., 2004). Studies indicate that scores of CL-FODS correlate negatively with religiosity and spirituality (Clements, 1998; Suhail and Akram, 2002; Mahboubi et al., 2014) and positively with neuroticism (Mooney and O’Gorman, 2001). Indeed, neuroticism (as a personality trait) is linked to generalized anxiety and preliminaries studies have demonstrated positive associations between neuroticism and death anxiety (Loo, 1984; Frazier and Foss-Goodman, 1989; Pradhan et al., 2020). Elsewhere, it has also been demonstrated that people with high spiritual well-being have low death anxiety (Routledge and Juhl, 2010; Lyke, 2013; MacLeod et al., 2019; Feng et al., 2021). Because spirituality offers sense of faith, meaning and purpose life and inner peace, people with high spirituality are less afraid of death and more prone to accept death. Neuroticism and spiritual well-being was used in the present study to test the concurrent validity of the CL-FODS.

Finally, CL-FODS has been validated in a number of cultural backgrounds thus testifying to adequate psychometric aspects (Abdel-Khalek and Lester, 2004; Tomás-Sábado et al., 2007; Mosaku and Ajenifuja, 2008; Venegas et al., 2011; Júnior et al., 2018; Bužgová and Janíková, 2019). The availability of valid measures addressing fears of death is essential, but no study has investigated a French version of CL-FODS. Therefore, the present study aimed to adapt the translated revised Collett-Lester Fear of Death Scale (CL-FODS, Lester, 2004) in a general French sample and to determine its psychometric properties. The validity of the fear of death construct was analysed through exploratory factorial analysis (EFA) and confirmatory factorial analysis (CFA). Convergent validity of the four subscales of the CL-FODS was assessed by comparing these to the Templer Death Anxiety scale and concurrent validity was assessed through correlating the CL-FODS with neuroticism) and spirituality.

## Method

### Participants and Procedure

The study involved 590 French voluntary participants from different regions of France. Web-based strategies were used to recruit our volunteers including emails, invitations via personal and professional networks and posts on forums in which individuals with shared interests could join the study. Study enrolment took place entirely online through a data collection software program, between May and July 2019. The study questionnaire took approximately 10/15 min to complete. Before proceeding, all of the volunteers who participated in the survey provided informed consent and were assured of anonymity and confidentiality.

The experimental protocols were approved by the local research ethics committee (CER-TP – n° 2020-10-06).

### Measures

Socio-demographic characteristics of the participants including gender, age, relationship status, socio-professional category, education and religious affiliation were recorded.

#### The Revised Collett–Lester Fear of Death Scale (CL-FODS)

The Revised Collett–Lester fear of death scale (CL-FODS) was used to assess fear of death experienced by respondents (Lester, 2004). The CL-FODS consists of 28-items which assess attitudes toward death, and the process of dying for both oneself and others. The items are organized in four subscales: (1) fear of one’s own death; (2) fear of the process of one’s own dying; (3) fear of the death of others and (4) fear of the process of others dying. Each of the four subscales contains seven items. The answers are given on a 5-point Likert-type scale (1 = not to 5 = very). Scores are obtained for the total scale and for each sub-dimension, calculating the average of the respective answers. The highest mean scores indicate greater fear of death or of the dying process.

#### The Translation Process of Collett–Lester Fear of Death Scale

In the present study, the CL-FODS was translated into French applying the back-translation procedure. Afterward, a committee of experts in death research (consisting of the authors of the manuscript, senior researchers, Ph.D. students and a clinical psychologist) inspected the translated version item by item to agree upon acceptable wording. The items retained are presented in Table 2. Finally, we pilot-tested the CL-FODS with 25 volunteer participants. All the participants stated that they had no difficulty understanding the items.

#### The Death Anxiety Scale

Anxiety in relation to death was evaluated with a French translation of the Templer unidimensional scale (Templer, 1970 – French translation: Lévy et al., 1982). This instrument consists of a 15-statement self-report questionnaire which uses a Likert response format on which the respondent must agree or disagree, by saying whether the statement is either true or false. Totalscores can range from 0 to 15, with higher scores indicating higher anxiety of death. The reliability coefficient in this study was 0.68.

#### Neuroticism

Neuroticism was assessed using the sub-scale of the Big Five Inventory composed of 8 items (John et al., 1991 – French validation: Plaisant et al., 2010). This instrument is a self-report inventory and respondents’ judgement was expressed on a five-point Likert scale, ranging from “1” (disagree) to “5” (agree). The reliability coefficient in this study was 0.84.

#### Spirituality

Spirituality was assessed using a Functional Assessment of Chronic Illness Therapy – Spiritual Well- a modified version for non-illness (FACIT-Sp-Non-Illness) (Peterman et al., 2002). The FACIT-Sp-Non-Illness is a 12-item questionnaire that measures spiritual well-being in a general population. This instrument is divided into three dimensions: Meaning, Peace and Faith. Respondents assess the items on a five-point Likert scale (from 0 “not at all” to 4 “very much”). The questionnaire provides four scores: one per dimension and one overall. A high overall score reflects a high level of spirituality. The reliability coefficient in our study was 0.86 for faith, 0.78 for peace and 0.72 for meaning dimensions.

### Statistical Analysis

To analyze the factorial validity of the revised CL-FODS, we randomly divided the study sample into two subgroups (sample 1, *N* = 207, sample 2, *N* = 383). With the first sample, we conducted an exploratory factorial analysis (EFA) using Varimax rotation. The subject-to-item ratio was 7.39 comprising a recommended ratio of between 5:1 and 10:1 (Osborne and Costello, 2004). To test the dataset for factor analysis suitability, the Kaiser-Meyer-Olkin (KMO) measure and Barlett’s test of sphericity were assessed. Because Kaiser’s eigenvalue (> 1) seems to be problematic and inefficient to determine the number of factors, parallel analysis were used to determine the number of factors (O’Connor, 2000; Ledesma and Valero-Mora, 2007). Items with a loading smaller than 0.50 or with loadings of 0.50 or greater on more than one factor were deleted. To confirm the factor structure of CL-FODS, a confirmatory factor analysis (CFA under the maximum likelihood method) was performed on the second sample (*N* = 383). To assess model fit, we examined normed chi-square (χ2/df), Comparative Fit Index (CFI), Tucker-Lewis index (TLI) and root mean square error of approximation (RMSEA). The following criteria were used: χ2/df ≤ 3, CFI and TLI ≥ 0.90 and RMSEA ≤ 0.08 with 90% confidence interval and standardized root mean square residual (SRMR) ≤ 0.06 (Hu and Bentler, 1998; Schermelleh-Engel et al., 2003). Once the factor structure of the revised CL-FODS was established, internal consistency was evaluated. Using the full sample (*n* = 590), Pearson’s correlations were used to evaluate convergent and concurrent validity between the CL-FODS subscales and death anxiety scale, neuroticism and spirituality. Finally, Pearson’s correlations were also used to examine links between CL-FODS and age and gender. Analyses were conducted using Mplus version 7.3 and additional data analyses were conducted using SPSS version 22.0.

## Results

Descriptive characteristics of the 590 participants are presented in Table 1. The average age was 41.6 (*SD* = 16.42) ranging from 18 to 89 years old, and there were 455 (77.1%) women and 135 (22.9%) men. 398 (67.5%) of the total sample were in a relationship and the majority of them had an education level of a Bachelor of Arts (BA) / Bachelor of Science (BSC) degree (27.6%; *N* = 163). In the sample, 47.6 % of participants were atheists (*N* = 281), 23.1% (*N* = 136) were non-practicing believers, 17.8% (*N* = 105) were agnostic and 11.5 % (*N* = 68) practiced a religion. Of those who said they were religious, 73.5% (*N* = 50) reported being Catholic. No significant differences were found between the two sample.

**TABLE 1:** Descriptive statistics of the demographic characteristics of participants.

Variables	Total sample (*N* = 590)	Split sample 1 (*N* = 207)	Split sample 2 (*N* = 383)
**Mean age (SD)**	41.6 (16.42)	43.7 (17.74)	40.5 (15.46)
**Gender N (%)**			
Female	455(77.1)	150 (72.5)	305 (79.6)
Male	135 (22.9)	57 (27.5)	78 (20.4)
**Civil status N (%)**			
In a relationship	398 (67.5)	139 (67.1)	259 (67.6)
Single	192 (32.5)	68 (32.9)	124 (32.4)
**Socio-economic classification N (%)**			
Self-employed (agriculture)	1 (0.2)	1 (0.5)	–
Small business owner and self-employed occupations	25 (4.2)	5 (5)	20 (5.2)
Large company employers, higher grade professional, administrative, and managerial occupations	188 (31.9)	57 (57)	131 (34.2)
Intermediate occupations	66 (11.2)	20 (9.7)	46 (12)
Skilled employees	105 (17.8)	41 (19.8)	64 (16.7)
Unskilled/semi-skilled employees	7 (1.2)	3 (1.4)	4 (1)
Students	127 (21.5)	38 (18.4)	89 (23.2)
Retired	58 (9.8)	34 (16.4)	24 (6.3)
Unemployed	13 (2.2)	8 (3.9)	5 (1.3)
**Educational levels N (%)**			
Elementary certificate	7 (1.2)	4 (1.9)	3 (0.8)
General certificate of secondary	17 (2.9)	6 (2.9)	11 (2.9)
Education or Youth training or BTEC	34 (5.8)	12 (5.8)	22 (5.7)
Baccalaureate	83 (14.1)	25 (12.1)	58 (15.1)
BTEC Higher national diploma	82 (13.9)	23 (11.1)	59 (15.4)
BA, BS/BSc or MS/MSc, MA	163 (27.6)	59 (28.5)	104 (27.2)
Master’s degree	156 (26.4)	56 (27.1)	100 (26.1)
Ph.D.	48 (8.1)	22 (10.6)	26 (6.8)
**Religious/spiritual affiliation N (%)**			
Atheist	281 (47.6)	103 (49.8)	178 (46.5)
Agnostic	105 (17.8)	36 (17.4)	68 (18)
Non-practising believer	136 (23.1)	42 (20.3)	94 (24.5)
Practising believer	68 (11.5)	26 (12.6)	42 (11)
Catholic	50 (73.5)	16 (61.5)	34 (81)
Muslim	9 (13.4)	4 (15.4)	5 (11.9)
Hindu	5 (7.5)	3 (11.5)	2 (4.8)
Protestant	2 (3)	1 (3.8)	1 (2.4)
Buddhist	2 (3)	2 (7.7)	–
**Neuroticism**	22.84 (6.71)	22.83 (1.09)	22.85 (0.45)
**Death anxiety scale (SD)**	7.10 (2.99)	7.18 (3.02)	7.07 (2.98)
**FACIT-sp-non-illness (SD)**	24.11 (6.32)	23.88 (1.02)	24.24 (0.83)
Peace	8.83 (2.18)	8.80 (2.32)	8.84 (2.11)
Meaning	9.48 (1.87)	9.60 (1.75)	9.41 (1.94)
Faith	5.80 (4.37)	5.47 (4.41)	5.93 (4.35)

### Factorial Validity of the Collett-Lester Fear of Death Scale

#### Exploratory Factor Analysis

First, for the first subsample (*N* = 207), the KMO measure was found to be 0.88 and Barlett’s test of sphericity was significant, with χ^2^ (378) = 2811; *p* < 0.001, thus fulfilling the prerequisites for conducting EFA. Exploratory factor analysis was conducted on the 28 items. The parallel analysis highlighted four factors to be extracted, while Kaiser revealed a five factor structure with an inflexion point after the fourth eigenvalue, supporting a four-factor solution. Results of EFA are presented in Table 2. The four factors explain 43.5% of the variance among the scale items (32.74%, 8.77%; 7.44%, and 5.18%). Factor 1 included the seven items of the “Own Death” subscale (eigenvalue = 9.17). Factor 2 included five items from the “Death of Others” subscale (eigenvalue = 2.45). Item 16 and 20 were the only items from the “Death of Others” not to load 0.50 or higher on this factor. Factor 3 included six items from the “Others Dying” subscale (eigenvalue = 2.08). Item 28 was the only item from “Others Dying” not to load on this factor. Factor 4 included five items from the “Own Dying” subscale (eigenvalue = 1.45). Item 12 and Item 14 were the only items from “Own Dying” not to load 0.50 or higher on this factor. The mean item-to-total correlations varied between 0.32 and 0.65, while the mean item-to-item correlations varied between 0.30 and 0.70. Cronbach’s alpha value was 0.84 for “Own Death”, 0.81 for “Own Dying” (0.82 when items 12 and 14 were omitted), 0.82 for “Death of Others” (0.82 when items 16 and 20 were omitted), and 0.83 for “Dying of Others” (0.84 when Q28 was omitted).

**TABLE 2:** Rotated Factor loading for the revised Collett–Lester fear of death scale (EFA – *n* = 207).

Items	Factor 1	Factor 2	Factor 3	Factor 4
**Own death**				
1. The total isolation of death (L’isolement total lié à votre mort)	**0.609**	0.271	0.024	0.195
2. The shortness of life (La brièveté de la vie)	**0.594**	0.217	0.086	0.151
3. Missing out on so much after you die [Passer à côté de tellement de choses une fois mort(e)]	**0.655**	0.287	0.020	0.179
4. Dying young (Mourir jeune)	**0.515**	0.339	–0.025	0.090
5. How it will feel to be dead (L’effet que cela ferait d’être mort)	**0.785**	0.105	0.198	–0.001
6. Never thinking or experiencing anything again (Ne plus penser ou ressentir quoique ce soit)	**0.691**	0.008	0.097	0.077
7. The disintegration of your body after you die (La décomposition de votre corps après votre mort)	**0.536**	0.046	0.191	0.127
**Own dying**				
8. The physical degeneration involved (L’implication de la dégradation physique)	0.178	–0.039	0.112	**0.696**
9. The pain involved in dying (La douleur impliquée dans vos derniers instants de vie)	0.210	0.383	0.079	**0.694**
10. The intellectual degeneration of old age (La degradation intellectuelle liée au grand âge)	0.068	0.008	0.164	**0.764**
11. That your abilities will be limited as you lay dying (Le fait que vos capacités seront diminuées sur votre lit de mort)	0.310	0.196	0.029	**0.677**
12. The uncertainty as to how bravely you will face the process of dying (L’incertitude quant au courage dont vous ferez preuve face à vos derniers instants de vie)	0.257	0.289	0.108	0.346
13. Your lack of control over the process of dying (Votre manque de contrôle face à vos derniers instants de vie)	0.390	0.267	0.072	**0.565**
14. The possibility of dying in a hospital away from friends and family (La probabilité de mourir à l’hôpital loin de votre famille / vos amis)	0.308	0.298	0.059	0.170
**Death of others**				
15. Losing someone close to you (Perdre un être cher)	0.171	**0.659**	0.196	0.213
16. Having to see the person’s dead body (Avoir à regarder le corps d’une personne décédée)	0.172	0.233	0.256	–0.026
17. Never being able to communicate with the person again (Ne plus jamais avoir la possibilité de communiquer avec cette personne)	0.132	**0.650**	0.324	0.134
18. Regret over not being nicer to the person when he or she was alive (Regretter de ne pas avoir été plus agréable avec cette personne de son vivant)	0.271	**0.620**	0.213	–0.060
19. Growing old alone without the person [Vieillir seul(e), sans cette personne]	0.087	**0.746**	0.164	0.154
20. Feeling guilty that you are relieved that the person is dead (Se sentir coupable d’éprouver un sentiment de soulagement après la mort de cette personne)	0.218	0.267	0.300	–0.070
21. Feeling lonely without the person [Se sentir seul(e) sans cette personne]	0.208	**0.734**	0.174	0.193
**Dying of others**				
22. Watching the person suffer from pain (Regarder la personne souffrir)	0.143	0.181	0.**757**	0.002
23. Having to be with someone who is dying (Devoir être aux côtés d’une personne lors de ses derniers instants de vie)	0.356	0.106	**0.644**	–0.062
24. Having the person want to talk about death with you (Le fait qu’une personne veuille parler de la mort avec vous)	–0.089	0.253	**0.603**	0.439
25. Seeing the physical degeneration of the person’s body (Etre témoin de la dégradation physique de la personne)	–0.024	0.111	**0.729**	0.411
26. Not knowing what to do about your grief at losing the person when you are with him or her (Ne pas savoir quoi faire du chagrin lié à la future perte de la personne lorsque vous êtes en sa présence)	0.204	0.382	**0.574**	0.112
27. Watching the deterioration of the person’s mental abilities (Etre témoin de la dégradation des capacités intellectuelles de la personne)	–0.034	0.188	**0.643**	0.442
28. Being reminded that you are going to go through the experience also one day (Garder à l’esprit que vous devrez vous-même traverser cette épreuve un jour)	–0.388	0.224	0.299	0.163
Eigenvalues	9.17	2.45	2.08	1.45
Percentage of the variance	32.74%	8.77%	7.44%	5.18%

*Bold values indicate loading > 0.50.*

#### Confirmatory Factor Analysis

Following the EFA results, we tested two models. The first model was the four factor structure of the CL-FODS (four factors with 28 items, seven per subscale). The second model was the four factor structure omitting the five problematic items (Items 12, 14, 16, 20, and 28) with seven items for “Own Death,” five items for “Own Dying,” five items for “Death of Others” and six items for “Dying of Others.” The goodness-of-fit indices of the CFA are presented in Table 3. The original four factor solution (Model 1) did not meet the criteria [χ2/df = 4.03; *p* < 0.001, CFI = 0.78, TLI = 0.76, RMSEA = 0.09 (0.08–0.09) and SRMR = 0.08]). Indices of Model 2 met the criteria for χ2/df, RMSEA and SRMR (χ2/df = 3.36; *p* < 0.001, RMSEA = 0.08 [0.07–0.09] and SRMR = 0.06), but CFI and TLI were lower than the expected thresholds (CFI = 0.86, TLI = 0.84). Inspection of the modification indexes suggested that the model would be improved by correlating a subset of error variances, and especially those of items *“5 and 6” and “19 and 21.”* The presence of correlated error may be due to a certain degree of overlap. Thus, a model was computed in which the error measures were freely estimated. The revised model had a satisfactory fit, as all the values were within the threshold of acceptability [χ2/df = 2.51; *p* < 0.001, CFI = 0.91, TLI = 0.90, RMSEA = 0.06 (0.06 – 0.09) and SRMR = 0.006]. The revised model was significantly better than the previous one *[Δχ^2^* (1) = 40.15, *p* < 0.001]. The standardized regression coefficients ranged from 0.43 to 0.86. Mean scores for subscales on the revised 23-items of the revised CL-FODS were 2.66 (SD = 0.97) for “Own Death,” 3.79 (SD = 0.86) for “Own Dying,” 3.81 (SD = 0.85) for “Death of Others,” and 3.23 (SD = 0.84) for “Dying of others.”

**TABLE 3:** Goodness-of-fit indices for the Revised CL-FODS four factor structure (*n* = 383 –CFA).

	Fit measures				
	χ^2^/df	CFI	TLI	RMSEA [90% C.I.]	SRMR
Model 1	4.03	0.78	0.76	0.09 [0.08-0.09]	0.08
Model 2	3.36	0.86	0.84	0.08 [0.07-0.09]	0.06
Model 2a	2,51	0.91	0.90	0.06 [0.06-0.07]	0.06

*Model 1: four factor solution with 28 items (7 items per subscale) / Model 2: four factor solution (23 items) / Model 2a = Model 2 with correlations between error variances.*

##### Convergent Validity

Convergent validity of the four subscales of the CL-FODS was assessed by comparing these against the Death Anxiety Scale (DAS) as the criterion measure. As shown in Table 4, scores on DAS were significantly positively correlated with subscale scores of the CL-FODS: “Own Death,” “Own Dying,” “Death of Others,” and “Dying of Others” (*r* = 0.65, *r* = 0.41, *r* = 0.39, *r* = 0.39, respectively; *p* < 0.001).

**TABLE 4:** Correlations between the Revised CL-FODS, Death Anxiety Scale (DAS), Neuroticism, and Spirituality (FACIT-Sp).

Measures	1.	2.	3.	4.	5.	6.	7.	8.	9.	10.
1. DAS										
2. Neuroticism	0.37***									
3. Spirituality (FACIT-Sp)	−0.14**	−0.26***								
4. Meaning (FACIT-Sp)	–0.06	−0.19**	0.53***							
5. Peace (FACIT-Sp)	−0.18***	−0.28***	0.71***	0.44**						
6. Faith (FACIT(Sp	–0.09	−0.18**	0.85***	0.12**	0.34***					
7. CL-FOLDS	0.60***	0.36***	−0.22***	–0.03	−0.20***	−0.22***				
8. Own Death	0.65***	0.29***	−0.18***	0.01	−0.17**	−0.19**	0.75***			
9. Own Dying	0.41***	0.22***	−0.12**	–0.03	−0.14**	−0.12**	0.76***	0.45***		
10. Death of others	0.39***	0.32***	−0.21***	–0.06	−0.18***	−0.23***	0.78***	0.46***	0.42***	
11. Dying of others	0.39***	0.26***	−0.17**	–0.07	−0.17***	−0.25***	0.76***	0.35***	0.35***	0.54***

*p < 0.05 - ** p < 0.01 - ***p < 0.001.

##### Concurrent Validity

Two measures (neuroticism and spirituality) were used to assess the concurrent validity of the CL-FODS and its subscales. As shown in Table 4, there was a significant correlation with the CL-FODS and the neuroticism scale (*r* = 0.36; *p* < 0.001). Participants with higher mean scores of neuroticism had higher scores on all the subscales of CL-FODS. In addition, participants with higher mean scores of spirituality had lower fear of death (*r* = −0.22, *p* < 0.001). Respondents with higher Peace and Faith scores reported less fear of death (respectively, *r* = −0.20, *r* = −0.22, *p* < 0.001). In contrast, results failed to support the predicted relationship between the meaning in life dimension and fear of death.

### Correlations With Participant Characteristics

Concerning participant characteristics, there was a main effect of gender, with women showing greater fear of death than men on the scores of the CL-FODS (*r* = 0.12, *p* < 0.05). The results indicated that the women had a greater fear of their own dying and death of others than the men (*r* = 0.16; *r* = 0.17, *p* < 0.01). Furthermore, the CL-FODS was significantly correlated with age in the present sample, with older individuals scoring lower levels of fear of death than young respondents (*r* = −0.19, *p* < 0.01). The results indicated that there was a significant difference between age in “Own Death” (*r* = −0.29, *p* < 0.01) and “Death of Others” subscales (*r* = −0.24, *p* < 0.01) with older adults reporting less fear of death.

## Discussion

The purpose of this research was to translate and test the psychometric properties of the revised Collett-Lester Fear of Death Scale in a French sample. This study highlighted promising results of the internal consistency, structural validity as well as convergent and concurrent validity.

The scale confirmed a four-factor model of CL-FODS. Nevertheless, among the initial 28 items, 23 were selected for the final French version of CL-FODS suggesting that the CL-FODS might require further psychometric refinement. Nevertheless, the mean scores on the CL-FODS scale are consistent with earlier studies, as regards to both the range of scores and the factors covered by the four subscales, with the scores for “*Death of Others*” and “*Dying of Others*” being the highest and the scores for “*Own Death*” and “*Own Dying*” being the lowest (Mooney and O’Gorman, 2001; Tomás-Sábado et al., 2007; Bužgová and Janíková, 2019). In addition, CL-FODS demonstrates a high correlation with the death anxiety scale denoting the convergent validity of the scale.

When evaluating relationships between CL-FODS and other theoretically related constructs, we found that the neuroticism measure was positively associated with fear of death while spirituality measure was negatively correlated with fear of death. Previous studies regarding the association between fear of death and neuroticism have also demonstrated that fear of death is higher in participants with a general disposition to anxiety or fearfulness (Lester, 1990; Neimeyer, 1994). Indeed, neurotic individuals are the most vulnerable to experiencing increased fear of death because they report more distress, symptoms and pain than people who are content and emotionally stable (Awopetu et al., 2017). This indicates that the mechanisms necessary for coping with existential issues would be less accessible for people with high neuroticism. Concerning spirituality, we found positive associations between two sub-scales of spirituality and fear of death. Indeed, both “*Peace*” and “*Faith*” factors were positively correlated with all the dimensions of the revised CL-FODS suggesting that respondents who experience greater feelings of peace and faith are less concerned about death than the others. It has been demonstrated that spirituality provides responses to existential and death-related queries and offers individuals a sense of sureness and control that may protect against anxiety raised by the prospect of dying (Speck et al., 2004; Jolley et al., 2010). In contrast, the “*Meaning*” dimension was not correlated with the revised CL-FODS. These results are not consistent with the literature which indicates that people who report a high purpose and meaning in their life tend to fear death less and to have a more positive and accepting attitude toward it (Zhang et al., 2019; Ding et al., 2020). Previous cross-sectional studies conducted among different age groups highlighted that meaning of life increases with age (Reker, 1997; Nygren et al., 2005; Fagerström, 2010). Our large age group bracket can explain this contrast result.

Our results highlighted a gender difference with women having higher scores relating to fear of death than men. However, our findings do not provide insight as to whether women have more fear of death than men or whether it is simply that they feel freer to express it due to cultural patterns. Indeed, Chan and Yap (2009) argue that men like women have fear of death but censor it or deny it. Moreover, women are usually the primary caregivers for the dying which could explain why they are more anxious about death than their counterparts (Kastenbaum, 2000). Otherwise, age was found to be a significant correlate of fear of death which suggests that on aging adults find the resources necessary to deal more effectively with fear of death. This result is in line with previous studies (Neimeyer, 1994; Thorson and Powell, 1994; Zana et al., 2009) which explain that this age difference can be due to psychosocial maturity (Rasmussen and Brems, 1996), or can be due to growing levels of spirituality with age (Koenig, 2006; Park, 2009). Moreover, personal experiences related to death, retirement and raising a family have been dealt with and thus it is also possible that older individuals are more prepared regarding death and death-related issues (Wu et al., 2002).

Some limitations of the present study should be considered in terms of the interpretation of these findings. Firstly, the present sample cannot be considered as entirely representative of the French population. Indeed, the majority of our participants were women and most respondents had higher levels of education than average. Secondly, while the study represents an important initial consideration of the psychometric properties of the CL-FODS, this scale needs to be tested for its discriminant and predictive validity as well as test-retest reliability. Thirdly, questions about past experiences of death (i.e., experience of loss of loved ones, life-threatening events, poor health such as physical diseases and psychological problems) were not collected in our study. However, we can assume that such external factors could impact an individual’s representation of fear of death. Thirdly, questions about personal health (poor health such as physical diseases and psychological problems) and past experiences of death (i.e., experience of loss of loved ones, life-threatening events) were not collected in our study. However, we can assume that such external factors could impact an individual’s representation of fear of death.

Despite these limitations, we conclude that the revised Collett-Lester Fear of Death Scale presents good validity and internal consistency and can be used with French samples in research and clinical settings. This instrument may provide an opportunity for research to be conducted on cross-cultural comparisons, and future studies should be carried out on different populations and sociodemographic backgrounds (e.g. clinical groups, terminally ill patients and geriatric samples).

## Data Availability Statement

The raw data supporting the conclusions of this article will be made available by the authors, without undue reservation.

## Ethics Statement

The studies involving human participants were reviewed and approved by CER-TP. The patients/participants provided their written informed consent to participate in this study.

## Author Contributions

MC, GB, and NB conceived and designed the study. MC collected the data and wrote the first draft of the manuscript. MC and NB performed the analysis. All authors critically reviewed and approved the manuscript prior to submission.

## Conflict of Interest

The authors declare that the research was conducted in the absence of any commercial or financial relationships that could be construed as a potential conflict of interest.

## Publisher’s Note

All claims expressed in this article are solely those of the authors and do not necessarily represent those of their affiliated organizations, or those of the publisher, the editors and the reviewers. Any product that may be evaluated in this article, or claim that may be made by its manufacturer, is not guaranteed or endorsed by the publisher.
